# Differences in brain gray matter volume in patients with Crohn’s disease with and without abdominal pain

**DOI:** 10.18632/oncotarget.21161

**Published:** 2017-09-22

**Authors:** Chunhui Bao, Peng Liu, Yin Shi, Luyi Wu, Xiaoming Jin, Xiaoqing Zeng, Jianye Zhang, Di Wang, Huirong Liu, Huangan Wu

**Affiliations:** ^1^ Key Laboratory of Acupuncture and Immunological Effects, Shanghai University of Traditional Chinese Medicine, Shanghai, China; ^2^ Life Sciences Research Center, School of Life Sciences and Technology, Xidian University, Xi’an, China; ^3^ Outpatient Department, Shanghai Research Institute of Acupuncture and Meridian, Shanghai University of Traditional Chinese Medicine, Shanghai, China; ^4^ Stark Neurosciences Research Institute, Indiana University School of Medicine, Indianapolis, Indiana, USA; ^5^ Department of Gastroenterology, Zhongshan Hospital, Fudan University, Shanghai, China; ^6^ Department of Radiology, Shanghai Mental Health Center, Shanghai Jiaotong University School of Medicine, Shanghai, China

**Keywords:** magnetic resonance imaging, Crohn’s disease, gray matter, brain, pain

## Abstract

Increasing evidence indicates that abnormal pain processing is present in the central nervous system of patients with Crohn’s disease (CD). The purposes of this study were to assess changes in gray matter (GM) volumes in CD patients in remission and to correlate structural changes in the brain with abdominal pain. We used a 3.0 T magnetic resonance scanner to examine the GM structures in 21 CD patients with abdominal pain, 26 CD patients without abdominal pain, and 30 healthy control subjects (HCs). Voxel-based morphometric analyses were used to assess the brain GM volumes. Patients with abdominal pain exhibited higher CD activity index and lower inflammatory bowel disease questionnaire scores than those of the patients without abdominal pain. Compare to HCs and to patients without abdominal pain, patients with abdominal pain exhibited lower GM volumes in the insula and anterior cingulate cortex (ACC); whereas compare to HCs and to patients with abdominal pain, the patients without abdominal pain exhibited higher GM volumes in the hippocampal and parahippocampal cortex. The GM volumes in the insula and ACC were significantly negatively correlated with daily pain scores. These results suggest that differences exist in the brain GM volume between CD patients in remission with and without abdominal pain. The negative correlation between the GM volumes in the insula and ACC and the presence and severity of abdominal pain in CD suggests these structures are closely related to visceral pain processing.

## INTRODUCTION

Abdominal pain is an important clinical manifestation of Crohn’s disease (CD). Such pain occurs not only in approximately 50% to 70% of patients during acute inflammation [1] but also in a significant proportion of patients during clinical and/or endoscopic remission [2]. Abdominal pain in patients with CD in remission has been associated with emotional suffering, disability, and high medical costs. Limited treatment options are currently available for this pain [3], and understanding the neurophysiological underpinnings of pain is fundamental to the development of an effective treatment.

The pathophysiology of abdominal pain in patients with CD in clinical remission is multifaceted. In these patients, pain is related to a dysfunction in brain-gut interactions [4], low-grade intestinal inflammation [5], visceral hypersensitivity [6], and other related mechanisms. The sensitization of the central nervous system (CNS) and reorganization of brain areas might play a role in visceral pain processing in CD [3, 7]. Magnetic resonance imaging (MRI) has been used to assess the structural plasticity and reorganization of the brain under different pathological conditions. Previous MRI studies have demonstrated pain-related cortical changes in patients with chronic conditions, such as irritable bowel syndrome [8, 9], chronic pancreatitis [10], and trigeminal neuropathic pain [11]. To date, several studies [12–14] have demonstrated structural changes in gray matter (GM) in patients with CD. The GM volume [12], sub-cortical volume [13], cortical thickness [12–14], surface area [13, 14]and folding [14] of multiple brain regions were significant changed. In our previous study [12], we found that, compare with healthy controls (HCs), patients with CD showed significant changes in GM volumes of multiple brain regions involved in pain, emotion, and homeostasis, and specific altered profiles of GM volume correlated with disease duration. Although these studies revealed changes in the GM structures in various brain regions, and pain has been associated with GM loss in numerous studies, the specific patterns of altered GM in patients with CD with or without abdominal pain have yet to be delineated.

In this study, we hypothesized that the structural changes in GM vary between patients with and without abdominal pain and that sustained abdominal pain is associated with changes in brain GM in areas involved in visceral pain processing. Specifically, the aims of the study were (1) to determine the changes in GM volume in patients with and without abdominal pain and (2) to potentially correlate the GM structural changes in specific brain regions with pain severity.

## RESULTS

### HCs vs. patients with CD with or without abdominal pain

#### Clinical variables

Among the 47 quiescent patients, 21 (44.7%) experienced abdominal pain. The average pain score ranged from 1 to 5.4, with a mean ± standard deviation of 2.68 ± 1.18.

There were no significant differences among the three groups in gender, age, height or weight. No differences existed between the pain and pain-free CD groups in disease duration, Montreal classification, or concomitant medication. The anxiety and depression scores of Hospital Anxiety and Depression Scale (HADS; including HADS-A and HADS-D subscales) in the pain, pain-free and HC groups were significantly different (*P* < 0.01). The patients in the pain group had higher HADS-A and HADS-D scores than did the subjects in the HC group (*P* < 0.01). The patients in the pain-free group had higher HADS-A scores than those in the HC group (*P* < 0.05). No significant difference was observed between the pain group and the pain-free group. The patients with abdominal pain exhibited higher CD activity index (CDAI) scores and lower Inflammatory Bowel Disease Questionnaire (IBDQ) scores than those of the patients without abdominal pain (*P* < 0.01).

The platelet (PLT) level and erythrocyte sedimentation rate (ESR) were not significantly different between the pain group and the pain-free group, while the C-reactive protein (CRP) level was significantly different between these two groups (*P* < 0.01). The Crohn’s disease endoscopic index of severity (CDEIS) scores of all the patients were below 3, with a mean (min∼max) value of 1.24 (0∼2.6) for the pain group and 0.89 (0∼2.0) for the pain-free group. No significant difference was observed between the two groups (Table 1).

**Table 1:** Clinical demographics of the CD with pain, pain-free CD, and HC groups

		CD with Pain (*n =* 21)	Pain-free CD (*n =* 26)	HC (*n =* 30)	*P* value
Gender (male/female)		12/9	20/6	20/10	0.352
Age (years)		30.86 ± 6.99	29.77 ± 7.23	30.47 ± 5.93	0.850
Height (cm)		169.33 ± 8.71	170.69 ± 5.96	169.60 ± 7.68	0.794
Weight (kg)		55.88 ± 9.66	57.62 ± 9.37	59.10 ± 6.47	0.410
HADS-A		6.52 ± 3.75^**^	5.27 ± 2.66*	3.40 ± 2.01	0.001
HADS-D		5.90 ± 3.99^**^	3.65 ± 2.87	3.00 ± 1.72	0.002
Disease duration (months)		88.29 ± 52.76	68.77 ± 45.04	—	0.178
CDAI		93.86 ± 36.00^###^	44.52 ± 30.74	—	0.000
IBDQ		162.95 ± 29.74^##^	187.77 ± 18.76	—	0.003
PLT		229.81 ± 33.00	209.00 ± 49.63	—	0.106
ESR		12.37 ± 5.10	9.85 ± 5.70	—	0.123
CRP		5.46 ± 2.35^##^	2.92 ± 2.72	—	0.002
CDEIS		1.24 ± 0.76	0.89 ± 0.50	—	0.068
Montreal classification^Δ^					
Age at diagnosis	A1	0	4	—	0.074
	A2	20	22	—	
	A3	1	0	—	
Location	L1	3	7	—	0.606
	L2	4	5	—	
	L3	14	14	—	
	L4	0	0	—	
Behavior	B1	7	9	—	0.385
	B2	1	2	—	
	B3	6	7	—	
	B1P	0	4	—	
	B2P	1	0	—	
	B3P	6	4	—	
Concomitant medication		15	15	—	
5-Aminosalicylate		9	7	—	0.240
Azathioprine		3	7	—	
5-Aminosalicylate & azathioprine		3	1	—	

*P < 0.05 and **P < 0.01 compared with the HC group; ^##^*P* < 0.01 and ^###^*P* < 0.001, compared with the pain-free group; ^Δ^ Montreal classification: A1, below 16 years; A2, between 17-40 years; A3, above 40 years; L1, ileal; L2, colonic; L3, ileocolonic; L4, isolated upper disease; B1, non-stricturing, non-penetrating; B2, structuring; B3, penetrating; P, perianal disease modifier. CD, Crohn’s disease; CDAI, Crohn’s Disease Activity Index; CDEIS, Crohn’s disease endoscopic index of severity; CRP, C-reactive protein; ESR, erythrocyte sedimentation rate; HADS-A, Hospital Anxiety and Depression Scale anxiety score; HADS-D, Hospital Anxiety and Depression Scale depression score; HCs, healthy controls; IBDQ, Inflammatory Bowel Disease Questionnaire; PLT, platelet. Values are presented as the mean values (standard deviations).

The CDAI scores of all the patients were correlated with the CDEIS scores (r = 0.445, *P* < 0.01), CRP levels (r = 0.361, *P* < 0.05), PLT levels (r = 0.407, *P* < 0.01), and ESR levels (r = 0.431, *P* < 0.01).

### Differences in GM volume

**Figure 1:**
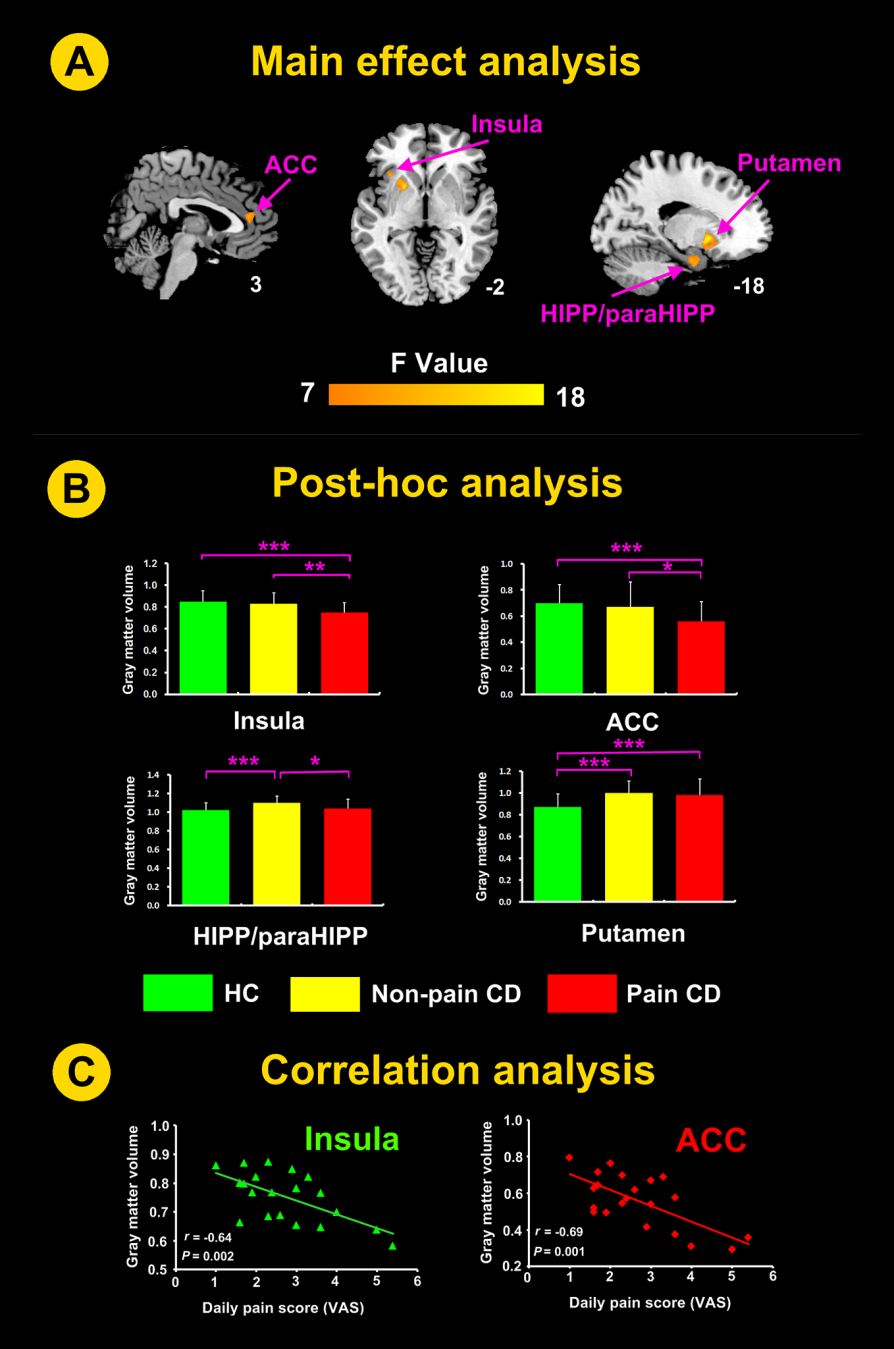
Significant differences in GM volumes in patients with CD-related pain, CD without pain, and HC groups with total GM volume, age, gender, weight, anxiety and depression as covariates (**A**) GM volumes of the putamen, HIPP/paraHIPP, insula and ACC differed significantly among the 3 groups. (**B**) Post-hoc analysis showed that the GM volumes of the insula and ACC in the CD with pain group were lower than the GM volumes in the remaining two groups. The GM volumes of the HIPP/paraHIPP in the pain-free group were higher than the GM volumes in the remaining two groups. The GM volumes of the putamen in the HC group were lower than the GM volumes in the remaining two groups, but the CD with pain and pain-free CD groups were not significantly different. (**C**) Correlation analysis between the GM volumes of the ROIs in the CD with pain group and the pain scores. ACC, anterior cingulate cortex; CD, Crohn’s disease; HC, healthy controls; HIPP/paraHIPP, hippocampal and parahippocampal cortex; L, left hemisphere; r, correlation coefficient; ROI, region of interest; VAS, visual analogue scale; *P < 0.05, **P < 0.01, ***P < 0.005.

A one-way ANOVA revealed that the GM volumes of 4 brain regions, including the left insula, hippocampal and parahippocampal cortex (HIPP/paraHIPP) and putamen, and right anterior cingulate cortex (ACC) (Table 2, Figure 1A), were significantly different among the pain, pain-free, and HC groups.

**Table 2:** Brain regions with significant differences in GM volume among the CD with pain, pain-free CD, and HC groups

Regions	Hem	BA	MNI			*F* value	Voxels
			X	Y	Z		
ACC	R	32	3	51	15	7.98	151
Insula	L	47	39	21	3	9.61	137
HIPP/paraHIPP	L	28	-18	–6	-24	10.09	178
Putamen	L	11	-24	15	-3	17.56	417

The results were based on the total GM volume, age, gender, weight, anxiety and depression as covariates. The statistical threshold was set at *P* < 0.05, and the false discovery rate was corrected for multiple comparisons. ACC, anterior cingulate cortex; CD, Crohn’s disease; HC, healthy controls; HIPP/paraHIPP, hippocampal and parahippocampal cortex; L, left hemisphere; R, right hemisphere.

The post-hoc analysis showed that the GM volumes of the insula and ACC in the pain group were lower than those in the pain-free and the HC groups. The GM volumes of the HIPP/paraHIPP in the pain-free group were higher than those in the pain and HC groups. The GM volumes of the putamen in the pain and pain-free CD groups were higher than those in the HC group; however, the difference between the two patient groups was not significant (Table 3, Figure 1B).

**Table 3:** The post-hoc analysis of the GM volume in the brain regions with significant differences among the CD with pain, pain-free CD, and HC groups

Regions	CD with pain (*n* = 21)	Pain-free CD (*n* = 26)	HC (*n* = 30)	Pain vs. pain-free		Pain vs. HC		Pain-free vs. HC	
				Effect size	*P* value	Effect size	*P* value	Effect size	*P* value
ACC_R	0.56 ± 0.15	0.67 ± 0.19	0.70 ± 0.14	0.64	0.02	0.96	0.002	0.18	0.432
Insula_L	0.75 ± 0.09	0.83 ± 0.10	0.85 ± 0.10	0.84	0.009	1.05	0.001	0.20	0.417
HIPP/paraHIPP_L	1.04 ± 0.10	1.10 ± 0.10	1.02 ± 0.08	0.70	0.018	0.22	0.295	1.06	0.000
Putamen_L	0.98 ± 0.15	1.00 ± 0.11	0.87± 0.12	0.15	0.619	0.81	0.003	1.13	0.000

Data are present as mean ± SD. ACC, anterior cingulate cortex; CD, Crohn’s disease; HC, healthy controls; HIPP/paraHIPP, hippocampal and parahippocampal cortex; L, left hemisphere; R, right hemisphere.

These results suggested that the patients with CD-related abdominal pain yielded the lowest GM volumes in the insula and ACC whereas the CD patients without abdominal pain exhibited the highest GM volumes in the HIPP/paraHIPP.

### Correlation between GM volumes and the severity of abdominal pain

The GM volumes of the insula and ACC were significantly negatively correlated with the daily pain scores in the patients with abdominal pain (r = -0.64, *P* = 0.002; r = -0.69, *P* = 0.001, respectively; Figure 1C). The GM volumes in the HIPP/paraHIPP of the CD patients with abdominal pain were not significantly correlated with the daily pain scores (r = -0.30, *P* = 0.18).

**Figure 2:**
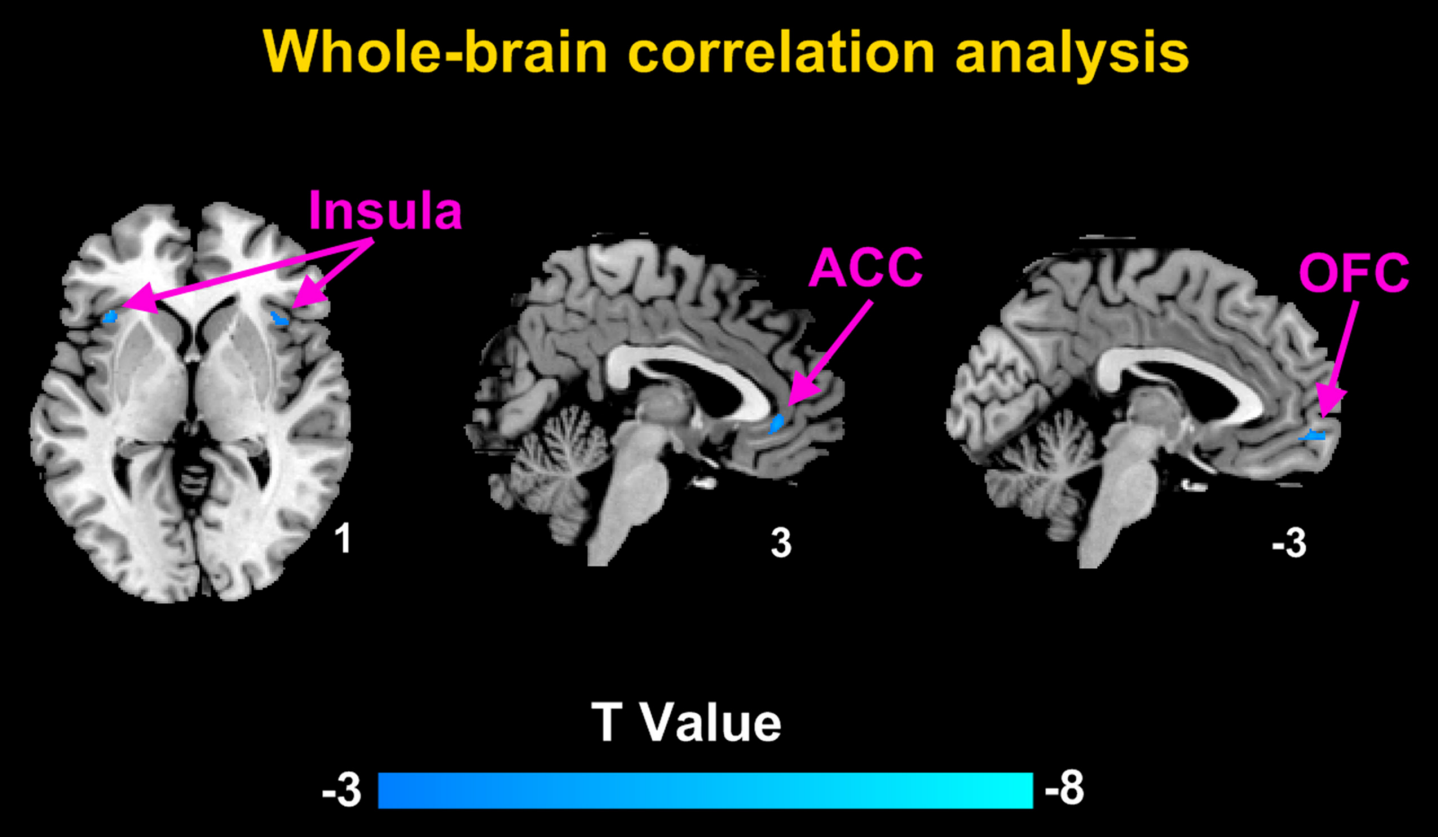
Brain regions with significant correlation between the GM volumes and daily pain scores in CD patients with abdominal pain using whole-brain correlation analysis The daily pain scores correlated negatively with the GM volumes of the right ACC, left OFC, and bilateral insula.

For the whole-brain analysis, the daily pain scores negatively correlated with the GM volumes of the right ACC, left OFC and bilateral insula (Table 4, Figure 2).

**Table 4:** Whole-brain correlation analysis between the GM volumes and daily pain scores in CD patients with abdominal pain

Regions	Hem	BA	MNI			*T* Value	Voxels
			X	Y	Z		
ACC	R	11	3	36	–3	–4.37	127
Insula	R	47	41	24	0	–4.86	134
Insula	L	47	–38	21	0	–5.56	120
OFC	L	11	–3	58	–9	–5.33	123

The results employed age, gender, weight, anxiety and depression as covariates. The statistical threshold was set at *P* < 0.005 (uncorrected) and cluster *P* < 0.05 (false discovery rate corrected). ACC, anterior cingulated cortex; BA, Brodmann area; Hem, hemisphere; L, left; OFC, orbitofrontal cortex; R, right.

## DISCUSSION

In the present study, we found the lowest GM volumes in the insula and ACC in remissive CD patients with abdominal pain. The reduced GM volumes in the insula and ACC were correlated with the daily pain scores of the patients. The patients without abdominal pain exhibited the highest GM volumes in the HIPP/paraHIPP. These findings suggested that the structural reorganization of the brain varied between CD patients with abdominal pain and those without pain, which may indicate a relationship between changes in specific brain structures and the existence of visceral pain in patients with intestinal inflammatory disease.

Similar to the observations in the patients with CD-related abdominal pain, abnormal changes in GM structures in the insula and ACC occur in patients afflicted with other chronic diseases such as lower back pain [15], irritable bowel syndrome [9] and fibromyalgia [16] and also similar to functional activity changes in these CD patients reported in our previous study [17]. These brain areas are important components of pain networks [18, 19]. The ACC and insula are the key components of the medial pain system, which might be primarily involved in processing affective-motivational aspects of pain. Studies [20, 21] have shown that the ACC is not only involved in the transmission of pain sensations but also plays a role in processing pain-related emotion. The ACC might respond to pain and participate in pain control [20]. The insula participates in the integration of visceral sensation and motor function and is responsible for transmitting pain signals to the frontal cortex [10].

The visceral pain (abdominal pain) signals may be transmitted to the "visceral and cortical pain matrix" via the brain - gut axis in CD patients [3]. Specifically, pain fibers carrying visceral nociceptive signals from the periphery synapse traverse through unmyelinated C fibers on both sides of the spinal cord to the dorsal root ganglia of spinal afferent nerves and synapse onto dorsal horn neurons and then ascend to the higher order brain processing areas in the thalamic nuclei and reticular formation [3]. The thalamic nuclei project to the primary and secondary somatosensory cortex where the sensory-discriminative aspects of pain is processed, and the reticular formation projects to the limbic system and frontal cortex, where the affective-motivational aspects of pain is processed [3, 20]. In this way, visceral pain signals in CD patients may be mainly transmitted to ACC and insula of the limbic system via the reticular formation.

We found that the GM volumes in the insula and ACC were negatively correlated with the daily pain scores in the patients with abdominal pain. The whole-brain analysis further confirmed the correlation and increased the reliability of the study. Structural GM abnormalities in the insula and ACC have been observed in nearly all pain-related diseases [22]. Similar negative correlations between GM volume and pain severity have also been observed in other imaging studies on GM plasticity, such as post-herpetic neuralgia, chronic back pain, and osteoarthritis [23]. The pain in CD patients in clinical remission is chronic, involves multiple factors. CD patients with abdominal pain yielded higher disease condition indices (CDAI scores and CRP levels) and significantly lower quality of life (QOL) than patients without abdominal pain, suggesting that the pain may not only be involved in the afferent processing of visceral pain signals, but may also be related to the stimulation of low inflammation in peripheral circulation.

In general, the mechanism underlying GM changes may be attributed to frequent and chronic nociceptive input and the subsequent functional reorganization and plasticity of the brain. The lower GM volumes could be related to a decrease in the size of neuronal somata, cell atrophy, or a decrease in intra-cortical axonal architecture (i.e., synaptic loss) [24]. In addition to the afferent processing of visceral pain signals, circulating inflammatory cytokines/mediators can reach the brain through circumventricular organs and active transport [25, 26], which may promote GM changes by inducing the apoptosis of neural or glial cells, including astrocytes and oligodendrocytes [27, 28], and activating endothelial cells, microglial cells, and macrophages. Furthermore, inflammatory cytokines/mediators may propagate signals within the brain by suppressing neurogenesis and exerting cytotoxic effects via increased glutamatergic activation and increased oxidative stress [28]. Thus, interactions among chronic nociceptive input, inflammation-induced neuronal changes, and brain reorganization may contribute to the reduced GM volumes in brain regions that are observed during pain processing and perception.

The patients without CD-related pain exhibited the highest GM volumes in the HIPP/paraHIPP, while the patients with CD-related pain exhibited lower GM volumes in this region. These findings suggest a role for specific brain structures in the control of abdominal pain in this disease: patients with the highest GM volumes exhibited better intrinsic pain relief (CD without pain) than patients with a lesser GM volumes increase (CD with pain). The HIPP/paraHIPP is associated with limbic and non-limbic system structures; the HIPP/paraHIPP regulates immune responses via the hypothalamic-pituitary-adrenal axis and neurohumoral pathways and plays an important role in neural immune regulation [29]. Studies have shown that experimental colitis and intestinal dysbiosis are associated with aberrant mRNA or protein expression of brain-derived neurotrophic factor in the HIPP/paraHIPP and the abnormal development of anxiety-like behavior [30]. The HIPP/paraHIPP may also interact with the vagus nerve, which is involved in the neural modulation of intestinal inflammation by releasing acetylcholine to regulate the functions of immune cells in the intestinal wall. In addition, an abnormal blood oxygen level-dependent (BOLD) signal has been found in the HIPP/paraHIPP in inflammatory bowel disease patients relative to control subjects [31]. Furthermore, given the critical role of the HIPP/paraHIPP in pain processing and modulation [32] and the finding that the highest GM volumes were present in the patients without abdominal pain may suggest an enhanced compensation by this region.

The present study has two limitations. First, this study was a cross-sectional study, which prevented the confirmation of a relationship between the abnormal GM changes in the CD patients in remission with the disease onset or the disease per se. In addition, neurotransmitter modulation and receptor binding in the associated brain areas and the correlation of these parameters with the GM structural changes must be elucidated. Future studies should observe longitudinal brain GM changes, together with the levels of related neurotransmitters in target brain regions, and to elucidate the neuroimaging biomarkers of CD.

In summary, our study represents the first investigation to demonstrate differences in brain GM volumes between CD patients in remission with and without abdominal pain. The reduced GM volumes in the insula and ACC in patients with CD with abdominal pain suggest a potential role in visceral pain processing and correlate with the presence and severity of abdominal pain. The findings may expand our understanding of the pathogenesis of CD, particularly the pathophysiology underlying pain in patients with CD.

## MATERIALS AND METHODS

### Subjects

All participants provided written informed consent. The study protocol was approved by the Institutional Review Board of Yueyang Hospital of Integrated Traditional Chinese and Western Medicine at Shanghai University of Traditional Chinese Medicine.

This study evaluated 21 patients with CD-related abdominal pain, 26 CD patients without abdominal pain, and 30 HCs. All the patients underwent a systemic and gastrointestinal examination, including a colonoscopy and pathological tissue biopsy. Laboratory tests and the colonoscopy were performed at 2 weeks and 1 month before the MRI scan, respectively. The CRP level, ESR, and PLT level were measured in all the patients. For the endoscopic examination, the CDEIS scores [33] were scored by an experienced endoscopist who was blinded to other details of the patients with CD.

The HCs were recruited via advertisements from Shanghai University of Traditional Chinese Medicine. These subjects were not treated with any medications, had no gastrointestinal symptoms or pain-related diseases, nor had negative findings in colonoscopic examinations.

### Eligibility criteria

The inclusion criteria were as follows: aged between 18 and 50 years, ≥ 6 years of education, right-handed, a CDAI of < 150, a CDEIS score of < 3 and in disease remission for > 12 months. Patients were excluded from the study if they met any of the following conditions: elevated biological disease activity indices (CRP > 10 mg/L, ESR > 20 mm/h, PLT > 300×10^9^/L); a history of CD-related abdominal surgery; treatment with corticosteroids, biological agents, psychotropic or opioid drugs in the previous 3 months; pregnant or lactating; a current or past history of psychiatric or neurological disorders, head trauma or loss of consciousness; claustrophobic; or had metal implants.

Furthermore, CD patients with chronic abdominal pain (i.e., dull periumbilical pain and/or pain in the right/left lower quadrant, etc. for ≥ 3 days per week for at least 12 months) were included in the pain group. Patients without abdominal pain for at least 12 months were included in the pain-free group.

### Symptoms and psychological assessment

The intensity of abdominal pain in the CD patients was evaluated using a 0-10 visual analog scale (VAS) [34]. The average pain score was calculated by dividing the total pain score by the number of days of pain during the week prior to the MRI scan. The CDAI [35] and the IBDQ [36] were used to evaluate the patients’ disease conditions and QOL, respectively.

The HADS [37] was used to assess the psychological status, including anxiety and depression, of all the subjects. The HADS included 14 questionnaire items and 7 items for each of the two sub-scales (anxiety and depression). Each item had a 4-point Likert scale (0-3 points) and a sub-scale of more than 8 points indicating anxiety or depression.

### Image acquisition

The MRI data were acquired in an interleaved multi-slice mode using a Siemens TRIO 3T clinical scanner (Magnetom Verio, Siemens, Erlangen, Germany). A high-resolution T1-weighted sagittal 3-dimensional magnetization-prepared rapid gradient-echo sequence was acquired for each participant with the following parameters: TR = 2300 ms; TE = 2.98 ms; field of view = 256 × 256 mm; matrix size = 256 × 256; in-plane resolution = 1 mm × 1 mm; slice thickness = 1.0 mm; flip angle = 9°; and slices = 176.

### Image analysis

#### Voxel-based morphometry (VBM) preprocessing

Structural image processing and analysis were performed using optimized VBM8 toolbox (C. Gaser, Department of Psychiatry, University of Jena, Germany; http://dbm.neuro.uni-jena.de/vbm8) in the Statistical Parametric Mapping software, version 12 (SPM12; Wellcome Trust Centre for Neuroimaging, University College London, England; http://www.fil.ion.ucl.ac.uk/spm), which was operated in a MATLAB (Mathworks, Natick, MA, USA) environment. All the structural images were transformed into a Montreal Neurological Institute (MNI) 152 standard space using linear transformations re-sampled to 1.5 × 1.5 × 1.5 mm^3^. The images were subsequently segmented into GM, white matter (WM), and cerebrospinal fluid (CSF). A modulation step was added to incorporate volume changes during spatial normalization [38]. This step involved multiplying each spatially normalized GM image by the relative volume of the image before and after normalization. The resulting GM images were smoothed with an 8-mm full-width-at-half-maximum (FWHM) isotropic Gaussian kernel.

### Statistical analysis

At the secondary level of analysis, we determined the differences in the GM volumes among the HCs and the patients with or without pain. A one-way analysis of variance (ANOVA) was performed at each voxel to assess the main effect, with a statistical threshold was set at *P* < 0.005 (uncorrected) and cluster *P* < 0.05 [false discovery rate (FDR) corrected]. The total GM volume, age, gender, weight, anxiety and depression were used as covariates.

For post hoc tests, regions of interest (ROIs) were selected from the sets of voxels within 6-mm spheres, with the centers at the peaks of the clusters with significant differences based on the ANOVA results of the VBM between the patients and the HCs. A 2-step correlation analysis was conducted in CD patients with abdominal pain: first, a Pearson’s correlation analysis was used to examine the relationship between the mean GM volume in each ROI and the pain score for the CD patients, with the total GM volume, age, gender, weight, anxiety and depression as covariates. The significance level was set at *P* < 0.05 with Bonferroni’s correction. Second, whole-brain correlation analysis was applied to determine potential correlations between the brain regions and pain condition. The significance level was set at *P* < 0.005 (uncorrected), and the FDR was corrected with the cluster size at *P* < 0.05.

The imaging results were overlaid on MRIcroN (http://www.sph.s.c.edu/comd/rorden/mricro.html) for presentation. The behavioral data were presented as the means ± standard deviations. The Statistical Package for the Social Sciences (SPSS) software package (SPSS Inc., Chicago, IL, USA), version 16.0, was used for the statistical analysis.
